# Machine Learning-Driven Precision Nutrition: A Paradigm Evolution in Dietary Assessment and Intervention

**DOI:** 10.3390/nu18010045

**Published:** 2025-12-22

**Authors:** Wenbin Quan, Jingbo Zhou, Juan Wang, Jihong Huang, Liping Du

**Affiliations:** 1 Food and Pharmacy College, Xuchang University, Xuchang 461000, China; 2 Collaborative Innovation Center of Functional Food by Green Manufacturing, Xuchang 461000, China; 3 School of Food Science and Engineering, South China University of Technology, Guangzhou 510641, China; 4 Rice Research Institute, Guangdong Academy of Agricultural Sciences, Guangzhou 510640, China; 5 College of Agriculture, Henan University, Kaifeng 475001, China; 6 Key Laboratory of Industrial Fermentation Microbiology Ministry of Education, Tianjin Key Laboratory of Industrial Microbiology, College of Biotechnology, Tianjin University of Science and Technology, Tianjin 300457, China

**Keywords:** machine learning, precision nutrition, dietary data, multi-omics, dynamic intervention

## Abstract

The rising global burden of chronic diseases highlights the limitations of traditional dietary guidelines. Precision Nutrition (PN) aims to deliver personalized dietary advice to optimize individual health, and the effective implementation of PN fundamentally relies on comprehensive and accurate dietary data. However, conventional dietary assessment methods often suffer from quantification errors and poor adaptability to dynamic changes, leading to inaccurate data and ineffective guidance. Machine learning (ML) offers a powerful suite of tools to address these limitations, enabling a paradigm shift across the nutritional management pipeline. Using dietary data as a thematic thread, this article outlines this transformation and synthesizes recent advances across dietary assessment, in-depth mining, and nutritional intervention. Additionally, current challenges and future trends in this domain are also further discussed. ML is driving a critical shift from a subjective, static mode to an objective, dynamic, and personalized paradigm, enabling a loop nutrition management framework. Precise food recognition and nutrient estimation can be implemented automatically with ML techniques like computer vision (CV) and natural language processing (NLP). Integrating with multiple data sources, ML is conducive to uncovering dietary patterns, assessing nutritional status, and deciphering intricate nutritional mechanisms. It also facilitates the development of personalized dietary intervention strategies tailored to individual needs, while enabling adaptive optimization based on users’ feedback and intervention effectiveness. Although challenges regarding data privacy and model interpretability persist, ML undeniably constitutes the vital technical support for advancing PN into practical reality.

## 1. Introduction

As research progresses, the understanding of the association between nutrition and health is deepening. Nutrients influence various physiological processes such as glucose metabolism, lipid metabolism, and amino acid metabolism [1], playing a crucial role in maintaining health and preventing diseases. Imbalanced nutrition intake can increase the risk of chronic diseases such as cardiovascular disease, diabetes, obesity, and cancer [2]. However, the current health of the population is concerning. A study in 2021 showed that there were 529 million people with diabetes worldwide, and the number is projected to exceed 1.31 billion by 2050 [3]. Recent research predicted that by 2035, the number of adults with a high body mass index (BMI > 25) will increase from 2.2 billion in 2020 to nearly 3.3 billion, and more than 39% of children aged 5–19 will be affected by high BMI [4]. The deterioration of health and the increasing prevalence of chronic diseases pose significant challenges to the global public health system, highlighting the inadequacy of current approaches to the prevention and treatment of diet-related diseases. Therefore, in recent years, an emerging nutrition management paradigm, ”Precision Nutrition (PN)”, has become increasingly imperative. As a developing concept in nutrition science, although a unified definition of PN remains pending, it is clear from the available definitions [5,6] that, compared with traditional nutrition strategies, PN emphasizes the precise assessment of individual health and nutritional needs by combining multi-dimensional data (e.g., diet, multi-omics, lifestyle, etc.) and the development of effective personalized nutritional intervention plans.

As one of the core data sources in the implementation of PN, dietary data can contribute to accurate evaluation of individual nutrient intake, profound elucidation of the mechanisms of the dietary impact on health, and the development of precise personalized dietary plans [7]. However, conventional dietary recording, analysis, and recommendation approaches are facing some challenges. For example, questionnaire-based approaches are frequently used in dietary records, but these methods are prone to various forms of errors, such as omission biases during manual documentation and temporal dietary fluctuations across diurnal cycles or seasonal variations [8]. These errors may lead to inaccurate dietary data collection and affect data quality. Besides, manual calculation and chemical analysis are commonly used for nutritional content evaluation of food [9], but are often affected by food quantification errors [10], leading to unreliable nutritional intake results. And the recommendations based on inaccurate assessments are usually ineffective and fail to keep pace with individual and environmental changes. Additionally, the heterogeneity and explosive growth of data have also hindered the application of traditional statistical analysis methods. Therefore, current dietary assessment and intervention approaches need urgent improvement to meet the demands of precision and dynamics in clinical conditions.

Machine learning (ML), with its ability to process high-dimensional data and capture nonlinear relationships [11], has provided robust support for data collection, processing, analysis, and application in PN, gradually becoming one of the core tools in this field. ML refers to the use of computational methods to enable computers to automatically learn patterns from reference data and/or previous iterations with mathematically describable principles, and to optimize performance for a specific task based on these patterns [12]. Although artificial intelligence (AI), machine learning (ML), and deep learning (DL) are often mentioned together, there are certain conceptual boundaries and technical differences among them. ML constitutes the foundational technology for achieving AI, while DL is a special and highly promising form of ML [13]. In this review, we primarily focus on the application of ML methodologies to advance PN. Therefore, our discussion in this article encompasses classical ML algorithms and DL architectures, both of which hold promising prospects in dietary assessment and intervention. According to the learning methods, ML can be classified into several categories, including supervised learning, unsupervised learning, reinforcement learning, and so on. When considering the specific learning tasks, ML can be categorized into various forms, such as regression, classification, clustering, and dimensionality reduction [14]. As for the evaluation of the ML models’ performance, different metrics can be used according to diverse factors such as tasks and practical significance. These evaluation metrics can reflect model performance from various dimensions. Common evaluation metrics for supervised learning are illustrated in Table 1, which will be frequently mentioned in the following parts. Traditional ML algorithms encompass linear regression, logistic regression, decision trees (DTs), support vector machines (SVMs), random forest (RF), gradient boosting methods (e.g., XGBoost), K-Nearest Neighbors (KNNs), and so on [15,16], which have been widely used for data analysis in nutrient estimation and diet analysis. Deep learning (DL) generally refers to ML methods based on multi-layer artificial neural networks. Common DL algorithms include convolutional neural networks (CNNs), recurrent neural networks (RNNs), restricted Boltzmann machines (RBMs), and autoencoders (AEs) [17]. Compared with traditional ML, DL demonstrates a superior capacity to process large-scale complex data and has been widely applied in fields such as natural language processing (NLP), speech recognition, and computer vision (CV) [18], thereby advancing food image recognition, food label data processing, and food nutrient composition evaluation [9].

Given the limitations of traditional dietary assessment and intervention, and the remarkable advantages of ML, it is particularly necessary to synthesize how ML is revolutionizing this field. Therefore, this review outlines an ML-driven nutritional management paradigm that is shifting toward precision, dynamism, and personalization, covering the entire pipeline from data acquisition and deep mining to intervention implementation. To highlight a focus, this paper concentrates on dietary data and provides a comprehensive overview of the advances in ML-driven research on diet and its interactions with other data sources across the above stages. Furthermore, we critically examined current challenges and proposed potential future directions in both technical and ethical aspects. Distinct from purely technique-oriented surveys, this review aims to serve as a bridge for cross-disciplinary practice (e.g., food science, nutrition, biomedicine, and data science), offering both academic context and practical reference for researchers from diverse backgrounds. It is designed to enhance the understanding of the current advancement and potential of ML in dietary assessment and intervention, thereby accelerating convergence and development in this field. This work is a valuable resource for catalyzing the translation of theoretical research into clinical practice.

## 2. Objective and Automated Dietary Assessment

**Figure 1:**
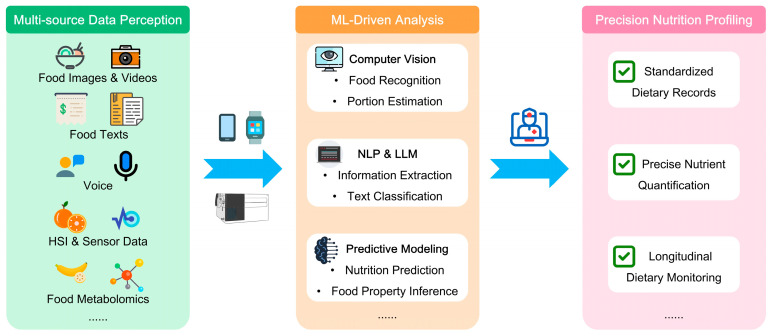
Machine learning-driven multi-source dietary assessment. ML: Machine Learning; NLP: Natural Language Processing; LLM: Large Language Model; HSI: Hyperspectral Imaging.

Comprehensive and accurate dietary data is the foundational pillar of nutrition management. It serves as a key reference for depicting individual differences in nutrient intake and validating the effect of diet intervention. However, as mentioned previously, self-reporting methods are sometimes so subjective that they can be affected by bias and errors, and impose burdens on participants. But the integration of ML technology has introduced transformative advancements in dietary data collection. With some advanced DL techniques, such as CV and NLP, various kinds of diet-related data, including food images and food labels, can be recorded and involved in research. ML is driving a paradigm evolution in dietary data acquisition, moving from subjective and slow self-reporting towards objective, real-time, automated, and multidimensional dietary monitoring (Figure 1). It will provide the indispensable data foundation for personalized and dynamic nutritional management.

### 2.1. Multimodal Dietary Data Acquisition

For research involving children or the elderly, questionnaire-based approaches often yield incomplete or erroneous data, making the acquisition of accurate dietary intake challenging [10]. Based on CV methods, especially CNN models, food image recognition technology can classify foods with greater precision, which enhances the automation of daily dietary recording and alleviates the load on participants and researchers. Wang et al. [28] proposed a food image retrieval and classification approach based on the Faster R-CNN network, which achieved a 5% improvement in classification performance compared to other models on the Dish-233 food dataset (233 dishes and 49,168 images). Combining food image recognition technology with wearable devices, long-term and passive food intake monitoring is gradually becoming a reality, thus significantly improving the feasibility and accuracy of dietary evaluation. Elbassuoni et al. [29] utilized a wearable camera coupled with an ML system to collect dietary data from children. Their framework deployed a wearable camera to capture images, and three ML models to identify food-related images, determine whether the image was actual food, and ascertain whether the food was consumed by the individual wearing the camera or by others, achieving an accuracy of 93.57% in practical application. It demonstrates that the incorporation of image acquisition from mobile cameras and CV image analysis is reforming the collection of dietary data by automating the recording process, which provides critical technical support for obtaining dietary data at a high spatial and temporal resolution.

After food classification from images, volume estimation is required to calculate nutrient intake. Konstantakopoulos et al. [30]’s system comprises a food classification module based on the EfficientNetB2 model (CNN) and a volume estimation module utilizing stereo vision techniques and algorithms. The volume module could estimate food volume with two food photos from smartphones via 3D reconstruction, achieving a MAPE of 10.5%. An application called Smart Diet Diary could estimate food volume from only one photo with the help of the GrabCut image segmentation algorithm, thereby enabling approximate caloric estimation [31]. But Smart Diet Diary required a coin as a reference object. Shao et al.’s study employed the RGB-D fusion network to integrate RGB and depth image information, enabling estimation of calorie, mass, fat, carbohydrate, and protein contents [32]. In their study, this framework achieved a percentage mean absolute error (PMAE) of 15% for calories. It illustrates that the multi-modal fusion approach can effectively compensate for the information limitations of single-modal approaches, thereby improving nutritional estimation accuracy.

However, it is critical to acknowledge that when applied to dietary information acquisition, CV techniques are facing certain limitations, including image quality and food databases. For instance, poor lighting [33] or food stacking [32] can significantly impair the effectiveness of image recognition. Furthermore, current publicly available food image datasets remain insufficient, since some studies even have to develop proprietary datasets to meet research needs [30,31]. Moreover, existing CV-based nutritional estimation frameworks often rely on simplified assumptions or static databases, which may overlook other contextual factors influencing nutritional composition, such as food brands and cooking methods. We will discuss how ML can incorporate these additional factors to advance the precision of nutrient intake estimation in the following parts.

In the data collection scenario, in addition to food images, a large amount of textual information, such as food labels or customer receipts, can be introduced to simplify the food classification process and enable precise dietary assessment, thereby reducing the requirements for researchers’ knowledge about nutrition and food. For example, Razavi et al. [34] employed ML algorithms, including DT, KNN, SVM, and neural networks, to predict unreported vitamin and mineral contents based on nutrient information from food labels. But such unstructured textual data is difficult to convert manually and efficiently into standard dietary records, due to brief and vague wording. With the help of the NLP technique, this extraction and linking process can be automated, transforming input texts into low-dimensional vectors. Hu et al. [35] used a pre-trained language model to process unstructured text from food labels, and combined it with supervised ML algorithms (Elastic net, KNN, XGBoost) to predict food categories and nutrition quality. Their pre-trained language model achieved an overall accuracy of 0.96 for food major category prediction and an R^2^ of 0.87 in predicting nutritional quality scores, both surpassing the performance of the bag-of-words method.

Since the popularization of ChatGPT, in recent years, large language models (LLMs) featuring more parameters and large-scale training datasets have aroused wide attention, which achieve better performance in various tasks, including entity extraction and machine translation [36]. Therefore, fine-tuned large LLMs are increasingly introduced to food and nutrition, facilitating new application directions such as mapping food texts to nutritional information. Assiri et al. [37] utilized LLMs such as GPT-4v, GPT-4o, and Gemini to extract nutritional information from food labels in English and Arabic, demonstrating reliable performance. The results of their study suggested that GPT-4o outperformed GPT-4v and Gemini. It highlights the necessity of developing multilingual dietary information acquisition tools to further enhance robustness in handling multiple languages and dietary cultures. With the advancement of multimodal fusion technology, more systems that integrate images, texts, and even voice are being developed for dietary recording and nutrition intake estimation. For instance, a vision-language model called UMDFood-VL integrated image and textual data to enable more rapid and accurate nutritional estimations, demonstrating a macro-AUCROC of 0.921 for lipid quantification [38]. Dodd et al. [39] provided a design for a semi-automated system for dietary assessment, based on image and voice records, which had been evaluated with the data collected in Cambodia. Although its automated transcription and translation performance only achieved an accuracy of 29.1%, this system still effectively reduced analysts’ workload, with Food Composition Database (FCD) matching accuracy reaching 77.2% after manual correction. Compared to text-based systems, this kind of voice-based system may be more suitable for regions with low literacy rates. Lo et al. [40] investigated the application of the multimodal foundation model GPT-4v in dietary assessment, finding that GPT-4v could integrate images before and after eating as well as text prompts to achieve dietitian-level performance in food recognition, portion estimation, and nutritional analysis. These advancements indicate that ML technologies are expanding the dimensions of dietary data collection. They not only effectively enhance the automation and precision of data processing but also address the limitations of single data source techniques.

However, we also observe that large models, designed primarily for general-purpose tasks, often exhibit biases or poor performance when applied to specialized tasks due to the lack of domain-specific training. For instance, without contextual cues, GPT-4v may misidentify banku (an African food) as rice or bread [40]. Additionally, DL-based models face persistent challenges such as high computational demands and poor model interpretability [41], which may hinder their integration into user-centric applications. Future research should prioritize exploring more efficient model architectures that balance performance with computational practicality.

### 2.2. Dynamic Nutrient Estimation: Transcending Static Tables

The food ingested by the body influences the human body in the form of nutrients, so precise estimation of the nutrient content in food is essential for revealing metabolic mechanisms and providing dietary guidance. It is worth noting that most dietary surveys relied on food composition tables or datasets for nutrient estimation, but these tables often ignore the influence of other factors such as season, origin, processing techniques, and cooking methods on nutrient content [8]. Consequently, these approaches may introduce some errors. For example, different varieties or cooking methods can significantly affect the nutrient content of Lentinus edodes [42]. Therefore, in recent years, ML models have been employed to predict the nutrient content of processed foods, which would contribute to the precision of nutritional intake evaluation and the improvement of food nutritional value. Muthukumar et al. [43] curated a dataset based on literature from Web of Science, Scopus, PubMed, and Google Scholar, and employed Support Vector Regression (SVR) and RF to forecast the protein content of plant-based foods, achieving the best Normalized Mean Squared Error (NMSE) of approximately 0.113. Naravane et al. [44] utilized various ML algorithms (Elastic Net, DT, RF, etc.) to predict vitamins and minerals in cooked plant-based and animal-based foods, and the results suggested that leafy greens and beef were the most predictable subcategories.

ML has also been integrated with non-destructive testing technology to predict nutrient contents of foods, enabling more precise nutrient intake estimation. For instance, hyperspectral imaging (HSI) offers both spatial and spectral information of food [45], and ML techniques have advanced the rapid and accurate analysis of hyperspectral images of food. The combination of HSI and ML has demonstrated capabilities in predicting nutritional values of various foods, such as beverages, meats, and grains [46,47]. Marín-Méndez et al. [47] predicted the nutritional values of 118 food samples from supermarkets, including mixtures of cooked meat and vegetables, via HSI systems with ML algorithms like Principal Component Analysis (PCA) and Ridge regression, suggesting the capacity for the analysis of complex foods. Furthermore, food metabolomics analysis can yield information regarding food composition, quality, and origin. Food metabolites may be related to human health-relevant nutrients, bioactive compounds, and metabolic pathways [48]. Food metabolomics coupled with ML enables more granular classification of food within the same category, facilitating differentiation of geographical origins [49] and processing methods [50]. Consequently, this approach can provide more detailed records of food information. The above technical approaches offer the potential for achieving dynamic assessment beyond food composition tables or food labels, and even extend the concept of “precision” from the individual level to specific foods. Nevertheless, although these technologies enable dynamic and precise dietary assessment, their integration into practical assessment may incur high costs and operational complexity. Thus, there is a need to strike a balance between high precision and low cost to facilitate the translation from the laboratory to clinical implementation.

## 3. Deep Mining of Dietary Data: From Data to Insights

**Figure 2:**
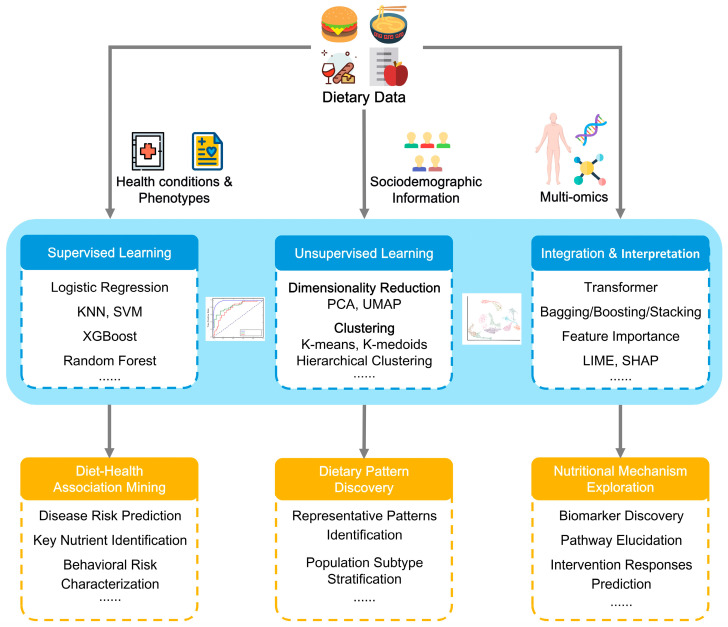
A framework for the deep mining of dietary data driven by machine learning. KNN: K-Nearest Neighbors; SVM: Support Vector Machine; XGBoost: Extreme Gradient Boosting; PCA: Principal Component Analysis; UMAP: Uniform Manifold Approximation and Projection; LIME: Local Interpretable Model-agnostic Explanations; SHAP: SHapley Additive exPlanations.

Raw dietary data merely reflect basic consumption facts. Only through systematic analysis can disparate dietary information be transformed into meaningful biological or clinical insights. With the continuous advancement of electronic devices and ML algorithms, more diet-related data can be recorded, rendering dietary data increasingly complex and high-dimensional. Traditional statistical analysis methods struggle to capture the intricate patterns and nonlinear relationships within such data. In contrast, ML is becoming a significant tool for deep mining of dietary data, facilitating the establishment of diet-health associations, the discovery of dietary patterns, and the exploration of nutritional mechanisms (Figure 2).

### 3.1. Diet-Health Associations Identification

While it is widely accepted that food intake is closely related to human health, such associations are often complex and difficult to uncover. Via supervised learning algorithms, chronic disease prediction is enabled based on nutritional intake, thereby identifying some significant dietary risk exposures, which can provide references for precise nutrition management. For instance, a cardiovascular disease (CVD) prediction model utilizing the XGBoost algorithm identified potassium, vitamin E, and vitamin C intakes as key micronutrients for predicting CVD risk [51]. Morgenstern et al. [52] constructed a CVD prediction model based on dietary data and general health information, achieving favorable predictive performance (AUROC = 0.821) and discovering an association between caffeine intake and increased CVD risk. In addition to CVD, ML has also been employed to evaluate the associations between dietary intake and diseases such as steatosis [53], undiagnosed hypertension [54], and premenstrual syndrome [55], which have identified critical eating behaviors related to disease progression, thereby offering references for health management. Despite these encouraging findings, a critical attitude remains essential. While ML helps mitigate the influence of covariates, it cannot establish causal relationships between nutrients and health outcomes. These results may exhibit context-dependent validity, as interactions among food components or changes in external environmental factors could significantly affect the reliability of observed associations.

### 3.2. Dietary Pattern Discovery

Although similar studies mentioned above have enhanced our understanding of the relationship between single nutrients and chronic diseases, in practice, nutrients and foods are rarely consumed in isolation, and various food components may interact in complex ways, exhibiting synergistic or antagonistic effects [56]. Consequently, in recent years, researchers have increasingly studied the relationship between diet and health from the perspective of dietary patterns. Dietary pattern refers to the combination and proportion of different foods and nutrients in the diet, encompassing rich dietary variables and exhibiting highly complex and nonlinear relationships [57,58]. Some unsupervised learning methods have been employed to identify representative dietary patterns from complex dietary data, aiding researchers in evaluating diets comprehensively. Silva et al. [59] applied the K-means clustering algorithm to identify two primary dietary patterns from dietary intake data and attempted to predict dietary patterns using six classification algorithms via socio-demographic and clinical data in a Brazilian population dataset: the Prudent pattern, characterized by higher intakes of fruits and vegetables, and the Western pattern, marked by higher intakes of refined grains, legumes, and red meat. A study compared the performance of different clustering algorithms (K-means, K-medoids, and hierarchical ward) in identifying dietary patterns for both sexes in the Dutch population, and the results suggested that K-means clustering was the optimal algorithm for dietary pattern analysis [60]. Besides, the NLP technique has also been introduced to extract more detailed dietary pattern information from unstructured food-related texts. Choi et al. [61] proposed a novel NLP framework to extract the dietary patterns of Korean adults, which could output the specific food compositions of patterns and reduce subjective bias.

The identified dietary patterns can be further correlated with health outcomes, which is conducive to the deeper analysis of the intricate relationship between diet and health, and guiding personalized dietary intervention strategies. Combining PCA dimensionality reduction and K-means clustering, researchers identified two Moroccan dietary patterns (‘prudent pattern’ and ‘dangerous pattern’) related to colorectal cancer (CRC), and logistic regression results indicated that the ‘dangerous pattern’ is associated with a higher risk of CRC [62]. Although PCA is commonly used for identifying dietary patterns, it has difficulty capturing the nonlinear relationships in real-world datasets. Researchers explored the correlation between dietary patterns and hypertension among adult Japanese males, but they employed a nonlinear dimensionality reduction method: Uniform Manifold Approximation and Projection (UMAP), which could reveal complex relationships in high-dimensional data [63]. However, it should be recognized that clustering-based identification methods may exhibit sensitivity to data quality, algorithm selection, and preset parameters, potentially leading to compromised robustness in results. A study analyzed the relationship between animal-sourced or plant-based dietary patterns and Type 2 diabetes via XGBoost and found that such relationships could be confounded by lifestyle variables [64]. This illustrates the importance of a comprehensive study design and the consideration of other interrelated factors in research. If sociodemographic characteristics and other confounding factors are not adequately controlled in the study, it may lead to misleading conclusions.

### 3.3. Nutritional Mechanisms and Biomarkers Exploration

The studies mentioned above established relationships between foods or dietary patterns and health outcomes, but such macro-level associations can only reveal whether diet affects health. In-depth food nutrition research necessitates an exploration of nutritional mechanisms to understand how food induces changes in health outcomes through biological pathways. Omics technology can provide multi-level biological information, such as genes, metabolism, and microorganisms, covering intricate biological processes between food and phenotypes. Hence, integrating dietary data with omics data facilitates the discovery of key molecular targets, signaling pathways, or metabolic changes caused by dietary interventions in the human body, thereby delineating the specific mechanisms through which nutrients influence human health. For example, a whole-blood transcriptomic analysis revealed a negative correlation between alcohol consumption and the expression of DOCK4 and SORT1 genes, and they were positively correlated with obesity [65]. Similarly, a plasma proteomics study across two cohorts identified 8 plant-based diet-protein associations and implicated 3 overrepresented pathways (complement and coagulation cascades, cell adhesion molecules, and retinol metabolism) [66]. In the large-scale and high-dimensional datasets from such a research strategy, ML can serve as a critical computational bridge, effectively capturing the subtle signals and complicated patterns.

Genomics analyzes DNA sequences representing the genetic information that governs cellular structure and function [67]. It plays a pivotal role in elucidating the impact of genetic variation on individual differences in dietary response and susceptibility to diet-related diseases. Park et al. [68] identified some single-nucleotide polymorphisms (SNPs) related to carbonated sugar-sweetened beverage (CSSB) intake via Genome-Wide Association Study (GWAS) and developed a CSSB consumption prediction model based on genetic, demographic, biochemical, and lifestyle factors through XGBoost and Deep Neural Network (DNN) with the AUROC values of 0.860 and 0.92, respectively. It indicates the interactions between genetic predisposition and dietary patterns. Transcriptomics is concerned with the RNA profiles of organisms, reflecting dynamic changes in gene expression [67]. It can capture changes in individual gene expression after dietary interventions, thereby enhancing the understanding of the molecular mechanisms of the relationship between diet and health outcomes. Oghabian et al. [69] utilized RNA-seq data from 281 individuals’ subcutaneous adipose tissue in an 8-month dietary intervention study and employed the SVM algorithm to construct a model predicting the diet-induced weight loss success based on differentially expressed genes and enriched pathways. Genes involved in triglyceride synthesis and elongation, including GPAM, DGAT2, and ELOVL6, were identified in their model, indicating that individuals with more active triglyceride synthesis in subcutaneous adipose tissue may face greater challenges in achieving long-term weight loss. This highlights the potential of ML to unveil the molecular mechanisms underlying health outcomes led by dietary intervention. Proteomics provides information on changes in protein levels, which aids in the exploration of the molecular mechanisms. Some biomarkers reflecting the body’s nutritional status can be identified, thus enabling rapid and efficient evaluation of the body’s nutritional needs. Mahdavi et al. [70] collected serum proteomic data from high and low dairy intake groups, and built a predictive model for serum vitamin C (VC) levels in hyperinsulinemic individuals using the KNN algorithm. Analysis of the plasma proteins in their model revealed that serum VC concentration was associated with immune response, lipid transport, and the complement and coagulation cascades, suggesting that VC may affect the immune response in hyperinsulinemic patients. It demonstrates that ML can help researchers to screen out significant proteins, thereby revealing potential links between nutrients, biological processes, and health outcomes, and providing new clues for mechanism research. However, due to data noise or research design, the integrated analysis of dietary data and omics data may sometimes identify numerous candidate biomarkers or pathways, but only a small number of them exhibit genuine relevance to the targeted biological mechanisms. Therefore, conclusions derived from such analyses require experimental validation in biological models to confirm their reliability.

Metabolomics focuses on the metabolites or small-molecule chemicals involved in metabolism [67]. Compared with other omics, metabolomics is more closely associated with an organism’s phenotype, so it can reflect body metabolic changes from the combined influence of genetics and environment, thereby elucidating the metabolic processes linking diet to phenotype. Kouraki et al. [71] conducted a 6-week randomized trial where participants supplemented their diets with either Omega-3 or inulin. In this study, various ML algorithms (e.g., elastic net, Random Forest) were employed to distinguish the Omega-3 and inulin groups based on serum or fecal metabolite changes. Serum metabolites CMPF and IPA, as well as fecal metabolite EPA, contributed significantly to their models, suggesting that Omega-3 and inulin improve metabolic health through different metabolic pathways in the human body. This demonstrates that ML can be used to identify metabolic differences led by specific dietary interventions, revealing how foods or nutrients affect biochemical processes in the body. The insights from analysis of in vivo metabolomics can then further assist in guiding the development and optimization of food ingredients and formulations that target specific metabolic pathways. Additionally, the effects of the same dietary intervention can vary considerably across individuals. Metabolomics can capture differences in metabolic profiles among individuals, and when combined with ML, it can predict individual responses to dietary interventions. For instance, a study constructed a QLattice model based on plasma and urine metabolomics to predict the success of weight loss in individuals undergoing the New Nordic Diet intervention, identifying adipate and arginine as potential contributors to body weight regulation [72].

Dietary biomarkers are quantifiable biological indicators of dietary intake or nutritional status [73]. Since metabolomics can provide a profile of small-molecule chemicals involved in metabolism [67], metabolomics also serves as a significant tool for the identification of dietary biomarkers. Hu et al. [74] constructed interpretable ML models to distinguish the intermittent fasting pattern and the control pattern via fecal metabolites. In this study, the Random Forest (RF) model was the most desirable model, and the SHapley Additive exPlanations (SHAP) analysis indicated that Ganoderenic Acid C might be a potential biomarker to distinguish the two dietary patterns. Through applying the least absolute shrinkage and selection operator (LASSO) and multiple comparative ML models, another study even developed a multi-biomarker panel for the Healthy Eating Index to assess individual adherence to the healthy diet pattern, including serum carotenoids and vitamins [75]. Moreover, ML models (RF and DT) have also been proven to predict Mediterranean diet consumption using plasma metabolomics from controlled feeding males, but other factors, such as gender, might influence the performance of ML models [76]. With the help of ML, metabolites can be further connected to chronic diseases and health outcomes, enabling early identification of individual health risks. Leiherer et al. [77] employed ML algorithms such as RF and SVM to predict the 4-year risk of type 2 diabetes in patients with cardiovascular risk using serum metabolomics data, realizing the maximum F1 score of 50%. These examples demonstrate that dietary biomarkers uncovered by metabolomics and ML have the potential to offer new ways for accurate dietary intake assessment and assist in evaluating disease progression and inferring pathogenesis. But most studies did not explore the dose–response relationship between dietary biomarkers and food intake, nor validate their specificity and sensitivity. These issues significantly limit the clinical applications of many biomarkers.

## 4. Personalized and Dynamic Dietary Intervention

Current general nutrition recommendations, such as dietary guidelines, are primarily based on scientific evidence of nutrition and human health and are proposed to meet the nutritional needs of the entire population [78]. However, these traditional recommendations tend to be relatively homogeneous. As more relevant research emerges, it has been found that dietary interventions and lifestyle choices have different impacts on individuals, likely due to differences in nutrient absorption and metabolism among individuals of various races, genders, genetics, and environments [79]. For example, the improvement effect of dietary intervention on cardiometabolic health has been shown to correlate with the tissue-specific phenotype of insulin resistance: individuals with muscle insulin resistance may benefit more from a high monounsaturated fatty acid diet, while those with liver insulin resistance may benefit more from a low-fat, high-carbohydrate diet [80]. This implies that individual nutritional needs are diverse, and traditional dietary guidelines may not be valid for all individuals. Effective dietary interventions require detailed customization based on individual conditions.

### 4.1. Personalized Intervention: Data Integration for Customization

Personalized dietary intervention customizes unique plans for different individuals by integrating data from various aspects. ML can facilitate the integrated analysis of these data, thereby enabling the consideration of more individual factors’ impact. Individual information, like Body Mass Index (BMI) and dietary preferences, can also serve as references for dietary recommendations. The dietary recommendation system of Nidhi et al. [81], based on K-means clustering, leveraged age, BMI, and dietary preferences as core user inputs. Their system categorized users into three groups: weight loss, weight gain, and health maintenance, and matched different dietary recommendations with varying calorie and protein ratios for each group. A hybrid diet recommender system incorporated the fat, carbohydrate, calorie, and protein contents of foods, as well as users’ BMI, into the considerations for food recommendations [82]. This system implemented K-means clustering to group foods with similar nutritional profiles and utilizes an RF classifier to recommend diets for users. The performance of this hybrid system outperformed existing models such as Multilayer Perceptron (MLP), RNN, and Long Short-Term Memory (LSTM). Apart from BMI, Basal Metabolic Rate (BMR) and Total Daily Energy Expenditure (TDEE) have also been employed in dietary guidance [83], which reflect individual calorie needs.

Chronic diseases are becoming an increasingly severe public health issue, so more dietary management systems that incorporate medical information have been developed in recent years to meet the nutritional requirements of patients. Vasuki et al. [84] developed a dietary guidance system for diabetic patients, using medical histories, dietary preferences, and blood glucose levels to offer personalized meal plans. Among three ML algorithms, including DT, RF, and Neural Networks, Neural Networks performed the best. A dietary recommendation system designed for hypertensive patients, via content-based filtering and the MLP algorithm, could provide dietary plans according to factors such as food preferences, allergies, smoking status, alcohol consumption, and blood pressure levels [85]. A study even developed a personalized health platform based on metabolomics that could analyze an individual’s risk of type 2 diabetes according to metabolite levels and provide recommendations regarding diet, exercise, and nutritional supplements [86]. There are also some systems that can recommend foods suitable for patients with diseases such as cancers [87] and polycystic ovarian syndrome [88], thus aiding in their dietary management. Although an increasing number of systems attempt to incorporate more aspects of information for better intervention, many studies treat different information as equally important [89]. This may contradict the actual situation, thereby affecting the applicability of these frameworks in clinical settings.

### 4.2. Dynamic Intervention: From Static Plans to Continuous Adaptation

Traditional dietary intervention usually formulates a fixed, periodic dietary plan based on a single medical consultation or pre-established rules. However, this static mode struggles to adapt to the dynamic changes in individuals over time, such as alterations in physical condition and lifestyle, leading to a gradual decline in intervention effect. Therefore, more emphasis should be placed on the dynamics of dietary intervention. It relies on continuous monitoring of users and integration of user feedback to adjust food plans promptly, thereby meeting users’ nutritional needs. Mogaveera et al. [90] proposed a health monitoring system that provides dietary and fitness recommendations. According to users’ basic information (height, weight, lifestyle, activity, medical conditions, etc.) and recent blood report (e.g., blood glucose, blood pressure, thyroid-stimulating hormone), their system employed the C4.5 decision tree algorithm to generate dietary and exercise advice, which outperformed the ID3 algorithm. Although this study achieves health monitoring by updating users’ latest reports, this approach lacks sustainability. It requires users to actively participate in medical examinations and update their information, which may lead to delays in disease management due to irregular intervals in information collection. In Jagatheesaperumal et al. [91]’s framework based on Internet of Things (IoT), sensors collected information such as blood oxygen level, pulse rate, and body temperature from individuals. Their framework implemented multiple ML algorithms (RF, CatBoost, Logistic Regression, and MLP) to assess individual health status, and then proposes dietary and health recommendations according to health risks. Among these, the CatBoost model demonstrated the best performance. This real-time monitoring enables early detection of health risks, facilitating long-term and dynamic dietary management. In addition to accessing real-time data streams, enhancing the adaptability of dietary recommendation models can advance precise and intelligent transformation in dietary interventions. Clarinda et al. [92] developed the AEPP (Adaptive Eating Pattern Predictor) by integrating ANN and Markov Chains. It provided personalized dietary advice via analyzing food intake and fitness data, with a feedback loop that adjusts suggestions based on ongoing user inputs and changing dietary patterns. The results of their study showed that AEPP outperforms DT, Gradient Boosting Machines (GBMs), and MLP in both accuracy and precision.

To adapt to the real-time changes in individual requirements and provide users with dynamic and adjustable diet plans, reinforcement learning has also been introduced to food recommendation. Reinforcement learning (RL) refers to an ML paradigm where an agent interacts with an uncertain and dynamic environment, continuously updates the parameters according to predefined objectives, and thereby identifies the optimal strategy to maximize the task rewards [93]. It exhibits significant advantages when dealing with complex and dynamic environments, such as low requirements for prior knowledge and adaptability to scenarios with sparse or delayed feedback [14,94]. Common RL algorithms include Q-learning, A3C, etc. [95]. RecipeRL is a multi-step food recommendation framework constructed through Markov Decision Process (MDP)-based Proximal Policy Optimization (PPO) policy [96]. RecipeRL achieved 94.68% precision and 95.67% normalized discounted cumulative gain (NDCG) in top-10 recommendation tasks, and maintained 93.2% precision and 95.71% NDCG even when user preferences dynamically changed, exceeding other algorithms’ performance significantly. Meal planning algorithm CFRL utilized the Deep Q-Network (DQN) RL algorithm and singular value decomposition (SVD) collaborative filtering approach, achieving superior user satisfaction compared to baseline methods in the generated meal plans [97]. RL introduces continuous decision-making and sustained interaction capabilities to food recommendation systems, enabling interactive dietary intervention. These systems can flexibly adjust dietary advice based on user feedback. However, the overall application of RL in dietary recommendations remains relatively limited, and rare research has incorporated other data sources, such as environmental information and food composition.

### 4.3. Interactive and Explainable Dietary Recommendation

Considering multiple types of data when providing food recommendations facilitates the comprehensive understanding of individual status and improves data reliability, thereby delivering precise dietary intervention strategies that meet personal needs. Therefore, some studies have explored integrating multimodal data into dietary recommendations. MyPlate application incorporated a food recognition module based on the Mask Region-based Convolutional Neural Network (Mask R-CNN) for automatic food identification and calorie calculation [98]. MyPlate’s recommendation module could generate dietary suggestions based on users’ historical intake records. Apart from dietary logs, multimodal data from individuals can also be integrated into dietary interventions. RecommenDiet system [99] could extract facial information with the pre-trained FaceNet model to predict height, weight, and BMI. These metrics were then used to calculate BMR and estimate calorie needs. The dietary plans were generated by KNN according to food preferences. Notably, FaceNet demonstrated superior performance for facial features extraction compared to CNN in RecommenDiet. RecommenDiet provides a convenient approach to infer physiological metrics, reducing reliance on user active self-reporting, but its dependence on facial images might raise privacy concerns. In addition, textual data, such as food descriptions, can also be introduced to the food recommendation to deliver more detailed and comprehensive information. The RecipeRadar system provided personalized recipe recommendations based on user inputs [100]. The core of RecipeRadar was a multi-input neural network model that categorized recipes using textual information (e.g., recipe names, ingredients, descriptions), and RecipeRadar also offered a chatbot for users to interact with. Rani et al. [101] integrated the RoBERTa model, a Transformer-based architecture, into the dietary recommendation system. This system could capture complex semantic information from food descriptions to ensure dietary advice aligns with user preferences. Although many systems have incorporated data such as images and text to deliver dietary recommendations based on calorie needs or user preferences, few interventions are guided by omics information, failing to fully account for individual heterogeneity. Furthermore, since not all users participate in dietary interventions merely for weight loss, future validation should incorporate diverse health phenotypes to verify the precision of the systems in health-oriented interventions.

Differing from traditional button-based interaction modes, LLMs can comprehend users’ natural language expressions, thereby enhancing the interactivity of dietary interventions. The framework of Aydın et al. [102] incorporated a local LLM (Mistral 7B), which could extract structured information from users’ natural language inputs, including dietary types, allergens, culture, and meal time. Then, their framework retrieved dietary suggestions based on nutritional goals and user-defined constraints from a filtered database. Traditional systems primarily rely on independent and static databases, whereas constructing knowledge graphs (KGs) can structurally connect knowledge across different entities, thereby enhancing interpretability. The KG-DietNet system contained a specialized KG based on multi-source data, including the U.S. Department of Agriculture (USDA) database, public food databases, brand products, and user historical dietary logs, which covered entities such as ingredients, nutrients, and health constraints [103]. With the help of a graph neural networks (GNN) and a LLM (e.g., GPT-4 or GPT-3.5 Turbo), KG-DietNet could generate personalized dietary plans. In this study, compared to systems relying solely on LLM, the KG module significantly improved nutritional fit and user satisfaction. In dietary interventions, few studies have compared the performance of different LLMs. Given the early accessibility and demonstrated capabilities in general domains, the ChatGPT series has been adopted in many recent studies for dietary intervention tasks like recipe generation and food recommender chatbots [104,105]. However, this does not imply its universal superiority for all nutrition-related tasks. A study indicated that Llama-3 (70B) outperformed GPT-4o in recognizing compound ingredients in dietary plans [106]. This underscores the growing need for specialized model evaluation in PN applications. Furthermore, research focusing on long-term clinical improvement from novel dietary intervention systems remains limited. Over extended periods, challenges such as concept drift [107] and sensor drift [108] may compromise model performance. Consequently, the real-world effectiveness of most food recommendation frameworks still requires rigorous validation.

## 5. Current Impact, Challenges, and Future Perspectives

**Figure 3:**
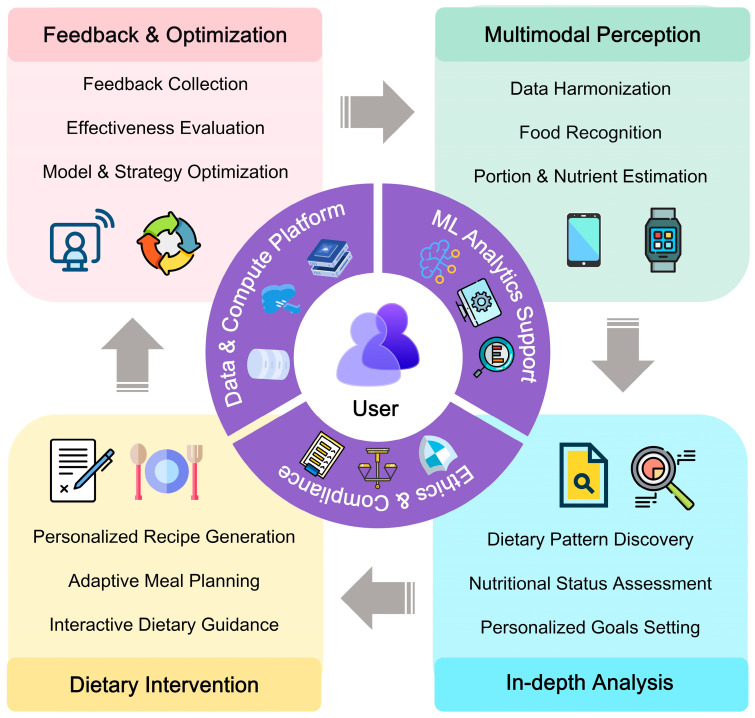
A machine learning-driven paradigm for precision nutrition management. ML: Machine Learning.

In summary, ML is fundamentally transforming traditional dietary practices (Table 2). It fuels multimodal data fusion, analytical depth, adaptive intervention, and iterative optimization, enabling a leap from generic recommendations to dynamic, personalized nutrition management through a loop system (Figure 3). Several studies have applied ML methods to real-world environments, achieving certain promising outcomes. Sun et al. [109] proposed an AI dietitian based on large language and image recognition models for type 2 diabetes management, which was developed into a WeChat Mini Program called FoodMed Companion. The results of preclinical validation suggested that GPT-4.0 successfully passed the Chinese Registered Dietitian Examination. In Sun et al.’s study, professional dietitians gave positive evaluations to most of ChatGPT’s responses to common nutritional questions (162/168), and the multi-label image recognition model achieved an average F1 score of 0.825. This highlights the significant potential of LLMs in expanding access to nutritional guidance. Meanwhile, automatic dietary assessment has been initially applied to clinical settings such as hospitals, simplifying the traditional, cumbersome dietary assessment process. Albaladejo et al. [110] conducted a pilot study to evaluate the effectiveness of an automatic image recognition device for recording food intake in hospital settings. The results of this study showed that the device was relatively accurate in estimating the weights of meat, fish, and starchy foods, but slightly less accurate for vegetables. The findings from pilot studies could provide valuable guidance for subsequent research. Moreover, some nutritional recording systems are attempting to integrate with smart devices for real-time nutritional monitoring. For instance, Montaina et al. [111] proposed a compact spectroscopic sensing platform that can be integrated into smart cups. Utilizing the KNN algorithm to analyze spectral data, this platform achieved an accuracy of over 96% in distinguishing common beverages. Such kind of solutions based on smart IoT devices hold significant potential, which can substantially reduce the time and effort required for users to manually input dietary information, thereby improving user adherence. However, most PN tools are still in the feasibility validation or pilot phase, and their long-term usability and user adherence need to be further verified in larger-scale and more realistic settings.

In addition, ML-based dietary interventions have also been implemented in clinical cohorts, exerting positive impacts on health status. Rein et al. [112] conducted a 2-week randomized crossover trial involving 23 patients with type 2 diabetes, who were blindly assigned to receive dietary interventions in a crossover sequence of either PPT (Personalized Postprandial Targeting)-MED (Mediterranean) or MED-PPT diets. The recommendations from PPT diets were individually tailored based on personal postprandial glucose responses (PPGRs) predicted by ML algorithms. The results of this crossover intervention showed participants exhibited significantly lower levels of glycemic measures during the PPT diet compared to the MED diet phase. The additional 6-month PPT diet of Rein et al.’s study demonstrated significant improvements in metabolic health parameters, including HbA1c, fasting glucose, and triglycerides, and 61% of the participants exhibited diabetes remission. Similarly, the results of a randomized controlled trial (RCT) demonstrated that, compared with standard type 2 diabetes care using traditional dietary interventions, personalized dietary interventions driven by ML and IoT significantly reduced levels of HbA1c, blood pressure, albuminuria, body weight, and BMI levels [113]. In this study, 72% of participants achieved diabetes remission with personalized interventions after 1 year. However, personalized dietary interventions do not always yield superior benefits compared with conventional dietary interventions. For example, there was no significant difference in weight loss efficacy between personalized diets and low-fat diets among adults with abnormal glucose metabolism and obesity [114]. This indicates that while PN interventions offer notable benefits, these are not universal. The effectiveness of PN implementation is constrained by factors such as intervention objectives and baseline characteristics. Thus, targeted optimization based on specific contexts is required before full-scale implementation.

Despite the prominent advantages, there are still some challenges that cannot be ignored. For instance, there is no denying that high-quality data is critical for developing robust ML models, but current publicly available databases in food and nutrition are still limited. This scarcity is particularly acute when studying rare cases or tasks. Many existing datasets are often limited to single regions, single food categories, or specific population groups [115], which may overlook the impact of other factors, such as culture and race. And the data structures from different sources, platforms, and institutions often lack a unified standard [116]. Consequently, models developed on specific datasets may not perform well in broader contexts and may require modifications to data preprocessing pipelines according to research objectives. And it is necessary to develop more databases concentrating on less prevalent regions or food types, and incorporating data of diverse types and sources. Besides, concerns regarding data privacy protection [117] also require attention, since relevant research often involves sensitive information such as genetics, environments, and medical records. The potential disclosure of such private information could exert a negative impact on research participants. Before large-scale deployment, it is imperative to ensure the use of data is both safe and compliant with regulations. External privacy training and external privacy self-assessment tools may contribute to privacy awareness and organizational resources for privacy protection, thereby reducing human-induced privacy breaches [118]. Deploying multiple privacy-preserving technologies (e.g., blockchain, federated learning, and cryptographic techniques [119]) may significantly enhance the protection of personal information.

In addition, the joint analysis and application of diverse data sources still need to be promoted. Introducing additional data sources can provide multi-level information, thereby gaining comprehensive insights and facilitating effective interventions. For instance, we observed that most nutritional assessment and intervention strategies frequently utilized body weight or BMI as primary health outcomes or reference parameters. In fact, weight and BMI have difficulty in accurately estimating individual fat content, which may potentially misclassify individuals with high muscle mass, such as athletes, as obese [120]. This illustrates that simple indicators sometimes have limitations in assessing the human health status and are not suitable as individual diagnostic tools. Other data sources, such as body composition, can serve as a more useful indicator for evaluating an individual’s health condition and monitoring the effectiveness of nutritional interventions. Therefore, the integration of more data sources, such as body composition, omics, and phenotypes, is essential for developing PN frameworks that enable precise risk stratification and adaptive intervention protocols.

Furthermore, the interpretability of ML models remains a limitation for clinical applications. Clinical practitioners not only desire ML models with superior performance but also require insights into the internal mechanisms of these models, such as the contribution of each feature to the output, since these insights can sometimes provide novel perspectives in research and enhance the credibility of models. Although most traditional ML models are relatively interpretable, these white-box models are often hard to achieve high performance in complex tasks, and interpreting DL models remains challenging (black-box models) [16], which partially hinders the clinical translation of research findings. Global surrogates, local interpretable model-agnostic explanations (LIMEs), and SHapley Additive exPlanations (SHAP) are some of the popular interpretability techniques [121]. SHAP can calculate approximate Shapley values and provide visualized global and local explanations [122], but SHAP may require extensive computational cost in high-dimensional datasets [121]. In dietary data analysis, SHAP is currently widely applied. Perhaps integrating professional knowledge into interpretability analysis would be beneficial to improve the correctness of ML explanations in specific domains.

We believe that with the assistance of ML, dietary assessment and intervention will evolve from the static, independent, and general-purpose model to a precise, dynamic, and intelligent paradigm in the future. As diverse modules are further integrated, this paradigm will be user-centric and integrate perception, assessment, and intervention into a holistic system. Multimodal data (e.g., images, texts, voice, and physiological signals) will be collected and transformed into structured information continuously and automatically. ML will serve as an effective tool to extract features from high-dimensional data and establish models, thus acquiring insights into individual health status and nutritional needs. ML-based systems can generate personalized and understandable dietary plans based on nutritional needs and individual contexts, continuously track user feedback, and adaptively optimize dietary strategies to ensure their effectiveness.

In the future, seamlessly integrating multiple data sources, such as food images, food labels, food nutrition knowledge, multi-omics, body composition, and environmental factors, into the framework will be a critical direction. These multi-dimensional data provide comprehensive information, thereby improving the accuracy of dietary assessment and the effectiveness of intervention. Besides, we also noticed that some LLMs targeting specific domains (e.g., question-answering [123], recipe retrieval [124], and nutrition estimation [125]) have been developed and demonstrated better performance than general models. These specialized LLMs can help researchers interpret literature, automate repetitive processes, and uncover scientific insights, thereby improving research efficiency. They can also popularize knowledge and generate personalized dietary plans for users, thus enabling health management. It is expected that in the future, more powerful specialized LLMs will be developed to address the challenges in dietary assessment and intervention. In addition, future efforts should concentrate on user satisfaction and potential ethical risks. Currently, many studies take health and user preferences as primary objectives, but excessive focus on these goals may cause models to inadvertently recommend extreme or monotonous dietary plans, thereby reducing user satisfaction and even inducing eating disorders. Some multi-objective optimization frameworks have attempted to simultaneously optimize multiple goals such as taste preferences, nutritional health, and dietary diversity [126,127]. More factors should be taken into consideration in future research to enhance practicality.

Crucially, if ML-generated dietary plans cause adverse effects on users, liability attribution can become controversial. Therefore, it is necessary to establish clear frameworks and regulations for responsibility determination and strengthen supervision in the future. Furthermore, as an interdisciplinary field, precise dietary assessment and intervention heavily rely on the contributions from researchers across food science, nutrition, biomedicine, bioinformatics, data science, and other related disciplines. Public users will increasingly engage in clinical practice. Therefore, more user-friendly tools and platforms need to be developed to reduce technical barriers and facilitate seamless connection between foundational research and clinical translation.

## 6. Conclusions

The global burden of diet-related chronic diseases and the limitations of traditional nutrition guidance underscore an urgent need for the implementation of PN. ML serves as a core catalytic force enabling a transformation across the entire dietary management pipeline. ML drives a critical evolution from a subjective and static mode to an objective, dynamic, and personalized paradigm. As detailed, CV and NLP automate the process and enhance the precision of dietary assessment, moving beyond self-reports. Through its capacity for high-dimensional data analysis, ML enables the discovery of dietary patterns and facilitates the elucidation of complex diet-health mechanisms. Furthermore, ML powers the development of dietary intervention frameworks that can tailor recommendations to individual profiles and dynamically adjust based on continuous feedback.

However, the translation of these technological advances into robust, equitable, and clinically effective solutions faces persistent challenges. These include data privacy, the need for diverse and high-quality datasets, model interpretability, and the practical barriers for the integration of precise high-throughput tools. Despite these hurdles, the potential impact of ML in PN is profound. The paradigm evolution will provide the indispensable technical foundation for moving from population-level guidelines to individual-level nutritional prescriptions. It will shift more focus from disease treatment to proactive health optimization, potentially reducing the long-term burden of diet-related chronic diseases on healthcare systems. Future progress hinges on interdisciplinary collaboration among clinicians, nutrition/biomedicine scientists, and data scientists.

## Figures and Tables

**Table 1:** Common evaluation metrics for supervised learning.

Task Type	Metric	Definition	Formula	Pros/Cons	Application Examples
Classification	Accuracy	Proportion of correctly predicted samples.	$Accuracy=\frac{T_{p}+T_{N}}{T_{p}+T_{N}+F_{p}+F_{N}}$	Fails in imbalanced classes	Exploration of the hypertension-nutrient intake relationship [19]
	Recall	Proportion of true positives among actual positives.	$Recall=\frac{T_{p}}{T_{p}+F_{N}}$	Recall and Precision are inversely related	
	Precision	Proportion of true positives among predicted positives	$Precision=\frac{T_{p}}{T_{p}+F_{P}}$		
	F1 score	Harmonic mean of precision and recall	$F1=2\times \frac{Precision\times Recall}{Precision+Recall}$	Balances precision and recall	
	Area under the Receiver Operating Characteristic Curve (ROC-AUC)	ROC curve plots True Positive Rate (TPR) vs. False Positive Rate (FPR). Higher values (closer to 1) indicate better performance.		Robust to class imbalance	
	Cohen’s Kappa coefficient	Agreement between a model’s predictions and true labels while accounting for random chance.	$Kappa=\frac{p_{o}-p_{e}}{1-p_{e}}$	Suitable for multi-class tasks	Prediction of malnutrition in children [20]
	Brier score (BS)	It quantifies probabilistic prediction accuracy. Lower values (closer to 0) indicate better performance.	$BS=\frac{\sum_{i=1}^{n}{\left(o_{i}-p_{i}\right)}^{2}}{n}$	Suitable for probability outputs	Prediction of vitamin D deficiency [21]
Regression	Mean absolute error (MAE)	Average absolute error between predicted values and true values	$MAE=\frac{\sum_{i=1}^{n}\left|y_{i}-{\hat{y}}_{i}\right|}{n}$	Less penalty on large errors	Prediction of human milk nutrient content [22]
	Mean absolute percentage error (MAPE)	Average relative error between predicted values and true values	$MAPE=\frac{\sum_{i=1}^{n}\left|\frac{y_{i}-{\hat{y}}_{i}}{y_{i}}\right|}{n}\times 100\%$	Undefined for zero true values	Prediction of minerals in tomatoes [23]
	Mean squared error (MSE)	Average squared error between predicted values and true values	$MSE=\frac{\sum_{i=1}^{n}{\left(y_{i}-{\hat{y}}_{i}\right)}^{2}}{n}$	Penalizes large errors	Prediction of snack fatty acid content [24]
	Root mean square error (RMSE)	Square root of MSE	$RMSE=\sqrt{\frac{\sum_{i=1}^{n}{\left(y_{i}-{\hat{y}}_{i}\right)}^{2}}{n}}$	Penalizes large errors; Matches the unit of the target	Prediction of food calories [25]
	Mean squared logarithmic error (MSLE)	Average squared difference between the logarithm of predicted values and true values	$MSLE=\frac{\sum_{i=1}^{n}{\left(\log\left(1+y_{i}\right)-\log\left(1+{\hat{y}}_{i}\right)\right)}^{2}}{n}$	Suitable for large value ranges	Prediction of epigallocatechin-3-gallate content in green tea [26]
	R^2^ (Coefficient of determination)	It measures how well the model fits the observed data. Higher values (closer to 1) indicate better performance.	$R^{2}=1-\frac{\sum_{i=1}^{n}{\left(y_{i}-{\hat{y}}_{i}\right)}^{2}}{\sum_{i=1}^{n}{\left(y_{i}-{\bar{y}}_{i}\right)}^{2}}$	Less effective for non-linear relationships	Neonatal parenteral nutrition management [27]

Note: $T_{p}$: True positive; $T_{N}$: True negative; $F_{p}$: False positive; $F_{N}$: False negative; $p_{o}$: Ratio of the sum of correctly classified samples in each category to the total number of samples; $p_{e}$: Sum of the product of true sample proportion and predicted sample proportion for each category; $o_{i}$: Observed label; $p_{i}$: Predicted probability; $y_{i}$: Observed value; ${\hat{y}}_{i}$: Predicted value; ${\bar{y}}_{i}$: Mean of observed values.

**Table 2:** Technical applications of machine learning in dietary assessment, data mining, and personalized intervention.

Task	Authors	Machine Learning Algorithms	Applications	Model Performance
Dietary Assessment	Wei et al. [28]	Faster R-CNN	Food image retrieval and classification	Accuracy = 0.754
	Elbassuoni et al. [29]	CNN	Recording children’s food intake	Precision = 82.23–93.57%
	Konstantakopoulos et al. [30]	EfficientNetB2	Automated image-based dietary assessment	Accuracy = 83.8%; MAPE = 10.5%
	Nadeem et al. [31]	Faster R-CNN	Food recognition & calorie calculation	Accuracy = 80.1%; Calorie error ≤ 10%
	Shao et al. [32]	RGB-D fusion network	Vision-based automated food nutrient analysis	Mean PMAE = 18.5%
	Razavi et al. [34]	RF, KNN, LR, Neural Networks, SVM, etc.	Predicting unreported micronutrient contents from food label information	R^2^ = 0.28 (for magnesium) −0.92 (for manganese)
	Hu et al. [35]	BERT, Elastic net, KNN, XGBoost	Food classification and calculation of nutritional quality using label information	Predicting food major categories: Accuracy = 0.98; FSANZ score prediction: R^2^ = 0.87 and MSE = 14.4
	Assiri et al. [37]	GPT-4v, GPT-4o, and Gemini	Extracting nutritional elements/values from food labels in English and Arabic	GPT-4o and GPT-4v: median Accuracy = 83–87% (English)
	Ma et al. [38]	CLIP, MLP	Estimating nutrient contents from food images and labels	Lipid quantification: macro-AUCROC = 0.921
	Lo et al. [40]	GPT-4v	Food recognition, portion estimation, and nutritional analysis	Food Detection: Accuracy = 0.936; F1 score = 0.946
	Muthukumar et al. [43]	SVR, RF	Predicting protein content in plant foods after processing	NMSE = 0.13
	Naravane et al. [44]	MLP, LASSO, Elastic net, Gradient boost, RF, DT	Predicting micronutrient contents in processed foods	R^2^ = 0.42–0.95
	Marín-Méndez et al. [47]	Multivariate linear regression, LASSO, Ridge regression, Elastic net, KNN, etc.	Predicting nutrition values of processed foods using hyperspectral imaging and machine learning	Near infrared (NIR): Protein: R^2^ = 0.88
Data Mining	Wang et al. [51]	XGBoost, KNN, RF, etc.	Cardiovascular disease risk assessment according to micronutrient intake	AUC = 0.952
	Morgenstern et al. [52]	Conditional Inference Forests	Developing the cardiovascular disease prediction model using dietary data	AUROC = 0.821
	Silva et al. [59]	K-means, SVM, KNN, etc.	Identifying dietary patterns and predicting them using sociodemographic data	Two patterns were identified; Accuracy = 69–72%
	Houwelingen et al. [60]	K-means, K-medoids, and hierarchical clustering; Naïve Bayes, KNN, RF, etc	Identifying and predicting dietary patterns in the Dutch population	K-means Clustering: Silhouette score = 0.045 (males) and 0.054 (females); Classification: Accuracy = 60–68%
	Choi et al. [61]	LDA	Extracting dietary patterns in the Korean population	Three patterns were identified.
	Noura et al. [62]	PCA, K-means, LR	Identifying CRC-related dietary patterns in the Moroccan population	Two patterns were identified. OR = 1.59
	Li et al. [63]	UMAP, K-means, LR	Assessing dietary patterns and their association with incident hypertension	Four patterns were identified. OR = 0.39 (Dairy/vegetable-based); OR = 0.37 (Meat-based)
	Eckart et al. [64]	PCA, XGBoost	Exploring Predictors of Type 2 Diabetes Within Dietary Patterns	Accuracy = 83.4%; AUROC = 68%
	Park et al. [68]	XGBoost, DNN	Exploring gene-diet interactions in carbonated sugar-sweetened beverage consumption and metabolic syndrome risk	AUROC = 0.860 (XGBoost) and 0.92 (DNN)
	Oghabian et al. [69]	SVM	Predicting weight loss outcomes using gene expression in subcutaneous adipose tissue	Max AUC = 0.74, 95% confidence intervals (CIs): 0.62–0.86
	Mahdavi et al. [70]	KNN, Linear regression	Predicting VC levels in hyperinsulinemia patients	Correlation coefficient = 68.5% ± 9.8%
	Kouraki et al. [71]	Elastic net, RF, LR	Comparing serum and fecal metabolomic changes after inulin or Omega-3 supplementation	Serum metabolites: Elastic net regression: AUC = 0.87; Fecal metabolites: Elastic net regression: AUC = 0.86
	Pigsborg et al. [72]	QLattice	Weight loss prediction after New Nordic Diet intervention	ROC-AUC = 0.81
	Hu et al. [74]	DT, KNN, RF, SVM, Naive Bayes	Exploring the potential biomarkers of intermittent fasting via metabolomics	Random Forest: AUC = 0.99, Accuracy = 0.94
	Liang et al. [75]	LASSO, Elastic net, the Gradient Boosting Regression, XGBoost, RF, MLP	Developing a panel of objective biomarkers that reflects the Healthy Eating Index	Primary multi-biomarker panel: RMSE = 10.10, R^2^ = 0.163 (LASSO, 10-fold cross-validation)
	Côté et al. [76]	RF and DT	Predicting Mediterranean diet consumption using plasma metabolomics	RF: Accuracy of 0.79 (95% CI: 0.71–0.86)
	Leiherer et al. [77]	RF, SVM, Naive Bayes, etc.	Predicting cardiovascular risk within 4 years using serum metabolomics	F1 score = 50%; Specificity = 93%
Dietary Intervention	Nidhi et al. [81]	K-means	Diet recommendation leveraging users’ age, height, BMI, and dietary preferences	Accuracy = 96%
	Vignesh et al. [82]	K-means, RF, MLP, RNN, LSTM	Automatic diet recommendation according to BMI and food preferences	Accuracy = 0.98
	Abdelmageed et al. [83]	RF, LSTM, KNN, SVM, etc.	Providing tailored nutrition advice with BMI, BMR, and TDEE	F1 score = 94%, and ROCAUC = 97%
	Vasuki et al. [84]	DT, RF, Neural Networks	Dietary guidance system for diabetes patients	Neural Networks: Accuracy = 0.90, F1 score = 0.90.
	Mogaveera et al. [90]	C4.5, ID3	Providing diet and exercise recommendations	Diet: Accuracy = 93.55% (C4.5, Prediabetes)
	Jagatheesaperumal et al. [91]	RF, CatBoost, LR, MLP	Providing diet and exercise recommendations based on health indicators	CatBoost: F1 score = 1
	Clarinda et al. [92]	ANN, MLP, DT, GBM	Providing personalized dietary recommendations by analyzing food intake and fitness data	Accuracy = 92.8%; Precision = 91.2%
	Liu et al. [96]	PPO, LGCN, SGNN, etc.	Interactive food recommendation	Precision = 93.2%; NDCG = 95.71%
	Amiri et al. [97]	DQN	Providing personalized and adaptable meal plans	Average Acceptance Score = 4.5/5.0
	Achuthan et al. [98]	Mask R-CNN	Food identification, portion analysis, calorie calculation, and food recommendation	Food Image Recognition: Average Precision = 0.825
	Pawade et al. [99]	FaceNet, KNN	Recommending diet plans based on facial features	Height, weight, and BMI estimation: RMSE = 6.2
	Aydın et al. [102]	RF, GB, Mistral 7B	Dynamic dietary recommendation	Calorie Prediction: R^2^ = 0.108; Structured Information Extraction: Accuracy = 91%
	Liu et al. [103]	GNN, GPT-4	Personalized dietary recommendation	Calorie Prediction: Percentage of error = 3.2%; User satisfaction: personalized alignment = 4.8/5.0

Note: Faster R-CNN: Fast Region-based Convolutional Neural Network; CNN: Convolutional Neural Network; RF: Random Forest; KNN: K-Nearest Neighbor; SVM: Support Vector Machine; XGBoost: eXtreme Gradient Boosting; BERT: Bidirectional Encoder Representations from Transformers; CLIP: Contrastive Language-Image Pre-Training; MLP: Multilayer Perceptron; SVR: Support vector regression; GB: Gradient Boosting; LASSO: Least Absolute Shrinkage and Selection Operator; LR: Logistic Regression; DT: Decision Trees; LDA: Latent Dirichlet Allocation; PCA: Principal Components Analysis; UMAP: Uniform Manifold Approximation And Projection; NMSE: Normalized Mean Squared Error; DNN: deep neural network; LSTM: Long Short-Term Memory; ANN: Artificial Neural Networks; GBM: Gradient Boosting Machines; PPO: Proximal Policy Optimization; LGCN: Localized Graph Convolutional Network; SGNN: Similarity-based Graph Neural Network; DQN: Deep Q-Network; Mask R-CNN: Mask Region-based Convolutional Neural Network; GNN: Graph neural network.

## Data Availability

Data will be made available on request.
